# Cell saver efficacy for coronary artery bypass surgery

**DOI:** 10.1186/1749-8090-8-S1-P106

**Published:** 2013-09-11

**Authors:** V Jovicic, S Putnik, A Djordjevic, M Cubrilo, D Terzic, N Aleksic, M Matkovic, D Cvetkovic, M Velinovic, M Ristic

**Affiliations:** 1 Clinic for Cardiac Surgery, Clinical Centre of Serbia, Belgrade, Serbia

## Background

Cell saver (CS) may be used during cardiac surgery to reduce and avoid allogeneic blood transfusion. The aim of the study was to determine how CS use during coronary artery bypass surgery (CABS) had an effect on haematological parameters changes before and after surgery, incidence and number of allogeneic blood transfusions and postoperative clinical complications and mortality.

## Methods

The study included sixty patients aged from 39 to 79 years presenting for CABS who were randomized to control or cell saver groups. We investigated blood parameters before and after surgery, ejection fraction, postoperative drainage, intraoperative parameters such as coronary artery bypass grafting type, number of graft, extracorporeal circulation and aortic cross clamping duration as weel as clinical complications and mortality.

## Results

There was no statistically significant difference between groups in haematological parameters changes before and after surgery (p>0.05). The CS use significantly reduced allogeneic transfusion requirements (p<0.05). Although there was less clinical complications in the group receiving CS, significant difference between groups in surgical outcomes that were presented as incidence of postoperative complications and mortality was not reached (p>0.05).

## Conclusion

The CS use during CABS reduced allogeneic transfusion requirements which are a great benefit in CABS. On the other hand, the effects of the CS on important outcomes such as postoperative clinical complications and mortality remain unproven.

